# Changes in white matter microstructure in the brain of patients with inflammatory bowel disease are associated with abdominal pain

**DOI:** 10.3389/fnins.2025.1570425

**Published:** 2025-07-08

**Authors:** Lingqin Zhang, Wuli Tang, Ling Yang, Xinyan Wu, Xin Deng, Li Yu, Yan Liu, Kang Li

**Affiliations:** ^1^Chongqing Medical University, Chongqing, China; ^2^Department of Radiology, Chongqing General Hospital, Chongqing, China; ^3^Department of Radiology, People’s Hospital of Chongqing Liangjiang New Area, Chongqing, China; ^4^Department of Radiology, Children’s Hospital of Chongqing Medical University, Chongqing, China; ^5^Department of Gastroenterology, Chongqing General Hospital, Chongqing, China

**Keywords:** inflammatory bowel disease, diffusion tensor imaging, brain white matter, negative emotions, multivariate analysis

## Abstract

**Introduction:**

In recent years, interest in the brain-gut axis has increased, and interactions between the brain and the gut may be closely related to recurrent clinical symptoms in patients with inflammatory bowel disease. We aimed to investigate the changes in white matter microstructure in the brain of patients and their relationships with clinical symptoms.

**Methods:**

A total of 96 patients with inflammatory bowel disease and 47 healthy controls were recruited for this study. All participants underwent diffusion tensor imaging of the brain. Tract-based spatial statistics were used to compare differences in brain white matter microstructure between the patients and healthy controls. Partial least squares correlation analysis was conducted to examine the relationships between changes in white matter microstructure in patients and their clinical symptoms.

**Results:**

Compared with healthy controls, patients with inflammatory bowel disease presented decreased mean diffusivity, axial diffusivity, and radial diffusivity in multiple white matter regions (*p* < 0.05, corrected). Further analysis revealed that patients with ulcerative colitis did not present significant differences in brain white matter microstructure (*p* > 0.05), whereas patients with Crohn’s disease presented abnormalities in multiple regions, including the corticospinal tracts, corona radiata, and corpus callosum. Multivariate analysis revealed that altered white matter in the brains of patients with inflammatory bowel disease was mainly positively correlated with pain-related negative emotions, such as scores from the fear of pain questionnaire and the pain anxiety symptoms scale.

**Conclusions:**

In patients with inflammatory bowel disease, particularly those with Crohn’s disease, alterations in the white matter microstructure that are primarily involved in pain processing have been observed.

## 1 Introduction

Inflammatory bowel disease (IBD) is a chronic inflammatory condition of the gastrointestinal tract that includes Crohn’s disease (CD) and ulcerative colitis (UC). It is typically characterized by recurrent abdominal pain, diarrhea, and blood in the stool (Chang, 2020). In addition, extraintestinal manifestations such as anxiety, depression, and fatigue can also occur, which seriously affects the quality of life of patients (Thomann et al., 2021). Long-term recurrent gastrointestinal and extraintestinal symptoms may lead to increased central noxious inputs, resulting in persistent neuroplastic changes in the brain microstructure of patients with IBD (Turkiewicz et al., 2021). These changes can further contribute to the development of irritable bowel syndrome (IBS)-like symptoms and exacerbate visceral pain (Agostini et al., 2023). However, although previous studies have revealed some associations between intestinal symptoms and extraintestinal manifestations and changes in brain microstructure in patients with IBD (Thapaliya et al., 2023), it is unclear which factor is most closely associated with brain changes.

The inflammatory state of the intestinal tract may mediate WM injury through the brain-gut axis by transmitting inflammatory mediators (Hung et al., 2023). For example, in an animal model, rats with necrotizing small bowel colitis developed brain WM injury associated with increased expression of the proinflammatory chemokines CXCL1 and CXCR2 (Yang et al., 2022). In patients with IBD, up to 42% of patients with CD and 46% of patients with UC show WM changes, whereas this proportion is only 16% in healthy volunteers, suggesting that white matter (WM) changes may be a common extraintestinal manifestation in patients with IBD (Andus et al., 1995). Notably, WM abnormalities in patients with IBD can lead to a decline in attention, memory, and executive function, as well as the development of mood problems such as pain, depression, and anxiety (Andus et al., 1995). Two previous studies (Hou et al., 2020; Zikou et al., 2014) reported that patients with IBD have altered WM fiber tracts, such as the corticospinal tract and superior longitudinal fasciculus, and these changes are associated with patients’ affective (anxiety, depression) and cognitive (verbal fluency language tasks) functioning.

However, previous studies (Chen et al., 2012; Hou et al., 2020) analyzing the associations between WM changes and clinical symptoms in patients with IBD have relied mostly on traditional univariate methods or basic multivariate models. While these methods can partially control for confounding variables, they have limitations with high-dimensional imaging data and complex behavioral indicators. For example, these studies fails to fully account for the interactions and intricate relationships among multiple variables and cannot address collinearity issues. In recent years, multivariate analysis methods have been widely used in neuroimaging research (Zhong et al., 2018). The method aims to find a spatial pattern from brain neuroimaging that can be used to differentiate between subjects’ behavior or grouping labels. Its effectiveness has been demonstrated by multiple studies, which have shown that brain spatial patterns can effectively classify and predict subjects’ brain activity, pain intensity, and cognitive abilities (Zhong et al., 2018; Ziegler et al., 2013). Compared with traditional univariate analysis methods, multivariate analysis methods can examine multiple behavioral and imaging indicators as a whole and can help identify interactions between multiple symptoms of IBD, thus constructing a more accurate and comprehensive correlation model between disease behavior and imaging.

In this study, we selected diffusion tensor imaging (DTI) as the primary neuroimaging technique because of its efficacy in assessing the integrity and alterations of WM microstructure. Specifically, DTI allows for the calculation of multiple metrics, including axial diffusivity (AD), mean diffusivity (MD), radial diffusivity (RD), and fractional anisotropy (FA), which collectively reflect the microstructural status of WM. We hypothesized that patients with IBD may have abnormal WM microstructures in the brain. The present study also aimed to identify key behavioral factors associated with WM changes. To achieve this goal, we compared WM microstructural changes between patients with IBD and healthy controls (HCs). Multiple symptom scales related to negative emotional states were incorporated for multivariate analysis. Additionally, partial least squares correlation (PLSC) analysis was used to examine the relationship between changes in WM and disease behavior in patients with IBD. This integrative approach aims to elucidate the WM neuropathological mechanisms linking WM abnormalities to the clinical manifestations of IBD.

## 2 Materials and methods

### 2.1 Participants

Ethical approval for the study was obtained from the Ethics Committee of Chongqing General Hospital (KY S2023-019-01). The committee also waived the requirement for informed consent. Patients with IBD diagnosed through a combination of imaging, endoscopy, pathology, and clinical manifestations were included in the study (Mowat et al., 2011). The inclusion criteria for the patient group were as follows: (1) right-handed patients; (2) complete clinical and imaging data; and (3) disease duration ≥1 year. The inclusion criteria for HCs were as follows: (1) right-handed individuals; (2) age-and sex-matched with the patient group; and (3) no chronic diseases or mental illnesses. Common exclusion criteria were as follows: (1) combined chronic pain disorders (such as fibromyalgia or chronic low back pain) diagnosed by rheumatologists using ICD-11 criteria or neurological/psychiatric disorders assessed by board-certified psychiatrists using the Structured Clinical Interview for DSM-5 (SCID-5, 2) history of brain disease or surgery confirmed by medical records and T1/T2-weighted magnetic resonance imaging (MRI) review; (3) contraindications to MRI such as metal implants and claustrophobia; and (4) pregnancy or breastfeeding.

### 2.2 Behavioral data acquisition

In this study, demographic and disease-related indicators of patients were collected using questionnaires. The demographic indicators included sex, age, disease duration, and education level. In the data collection process, standardized assessment criteria were used to determine disease activity: a clinical disease activity index (CDAI) ≥ 150 was used to define active CD, whereas a Mayo score ≥3 was used to define active UC. Disease behavioral indicators were mainly related to pain-related negative emotion scales, including the mean visual analog scale (Mean-VAS) and the max visual analog scale (Max-VAS) for the last 2 weeks and the fear of pain questionnaire (FPQ), pain anxiety symptoms scale (PASS), pain catastrophizing scale (PCS), pain sensitivity questionnaire (PSQ), and pain and vigilance awareness questionnaire (PVAQ). The gastrointestinal quality of life index (GIQLI) was used to assess patients’ quality of life comprehensively. In addition, anxiety-and depression-related indicators, including the Beck Depression Inventory (BDI) and State–Trait Anxiety Inventory (STAI), were collected. All questionnaires were completed on the same day that the patients underwent structural brain MRI.

### 2.3 MRI data acquisition

MRI scanning was performed using a 3.0 T MRI system produced by Siemens AG in Germany (MAGNETOM Skyra 3.0 T) with a 20-channel phased-array surface head coil for data acquisition. Prior to the scan, the patients were informed of the precautions for MRI scanning, such as removing any metal objects they were carrying. During the scanning process, the patients were instructed to close their eyes, remain awake, lie still, and avoid engaging in focused thinking activities. A sponge pad was used to stabilize the head and reduce head motion, and earplugs were used to reduce external noise. After preparation, the experimenters first acquired the patients’ whole-brain axial T1-weighted structural images. The scan sequence parameters were as follows: voxel size = 1 × 1 × 1 mm^3^, in-plane matrix size = 224 × 224, field of view = 230 mm, flip angle = 8°, number of slices = 144, slice thickness = 1 mm, and TR/TE = 2,200 ms/2.44 ms. Subsequently, the patients’ axial DTI images were acquired. The scan sequence parameters were as follows: voxel size = 2 × 2 × 2 mm^3^, in-plane matrix size = 104 × 104, field of view = 208 mm, number of slices = 60, slice thickness = 2 mm, TR/TE = 8,100 ms/86 ms.

### 2.4 DTI analysis

This study used the tract-based spatial statistics (TBSS) method to evaluate the integrity of participants’ brain WM. This method aligns each participant’s DTI image to a standard space and performs statistical analysis on the aligned images to extract microstructural features related to the specific research objective. These features include FA, MD, RD, and AD. TBSS can automatically analyze whole-brain WM without the need for predefined regions of interest and has been widely used in studies of WM (Croall et al., 2020). In this study, FSL (FMRIB Software Library) software was used for data processing and analysis of participants’ DTI images. The main steps included (1) removal of the skull for all patients, followed by removal of potential artifacts caused by magnetic resonance eddy currents and head motion; (2) calculation of DTI metrics such as FA for all patients; (3) alignment of DTI images of the patients to the standard template images; (4) averaging of the aligned images and calculating the whole-brain WM information; and (5) calculation of the FA, MD, RD, and AD of all of the patients on the WM microstructure. Following these computations, DTI parameters were obtained as neuroimaging indicators of WM for each patient.

### 2.5 Statistical analysis

SPSS 25.0 software was used to compare the demographic data between the IBD patient group and the HCs. For continuous variables that followed a normal distribution, the means ± standard deviations ($\bar{x}$ ± s) were reported and compared between the groups using *t*-test. For variables that did not follow a normal distribution, medians (interquartile ranges) were reported and compared using non-parametric tests. Categorical variables are presented as frequencies (percentages) and were compared using chi-square tests. To compare differences in WM between the two groups, a non-parametric randomized permutation test was used to compare DTI-related metrics in standard space, with multiple comparison correction using threshold-free cluster enhancement.

Furthermore, the study implemented PLSC for data integration. This multivariate approach systematically analyzed the relationships between behavioral measures and neuroimaging profiles across patients. Several studies have shown that the PLSC is well suited for multivariate analysis of various modalities in neuroimaging research, effectively extracting the correlations between brain neural structure or activity and behavior (Ziegler et al., 2013). In this study, the singular value decomposition method was used to implement PLSC, and the process consisted of four steps. First, the imaging matrix X and the behavioral matrix Y were standardized, and the covariance matrix R (R = X^T^Y) was calculated. Second, the covariance matrix R underwent singular value decomposition, expressed as R = U^T^SV. In this decomposition, the left singular matrix U represents the imaging significance matrix, the right singular matrix V denotes the behavioral significance matrix, and the matrix S is the singular value diagonal matrix, which reflects the correlation coefficients of the underlying factors. Next, the brain score matrix was obtained by multiplying the matrix X by U, and the behavior score matrix was derived by multiplying the matrix Y by V. The brain score matrix was correlated with the behavior matrix Y to obtain a behavior correlation coefficient matrix, which was used to characterize the extent to which the behavior variables contributed to the latent factors. Finally, a bootstrap sampling method was used to assess significance by calculating the bootstrap ratio to filter out the behavioral variables and brain regions with significant contributions.

## 3 Results

### 3.1 Comparison of general information

A total of 96 patients with IBD diagnosed at Chongqing General Hospital from September 2021 to September 2023 were included in this study. Among these patients, 64 had CD and 32 had UC. Additionally, 47 HCs matched for age and sex were recruited during the same period. Compared with the HCs, the patients with IBD had significantly higher scores on several negative emotion scales (including the Mean-VAS, Max-VAS, PSQ, BDI, and STAI), whereas the GIQLI scores were significantly lower (*p* < 0.05). The body mass index (BMI) of the patient group was greater than that of the control group (*p* < 0.05) (Table 1). The demographic, clinical, and psychometric profiles of the CD and UC subtypes were compared. Patients with CD were younger (*p* = 0.01) and had higher rates of surgical intervention (*p* < 0.001) and active disease (*p* = 0.01) than those with UC. In contrast, patients with UC had poorer GIQLI (*p* = 0.01). No significant differences were observed in pain perception or psychometric measures between the two groups (*p >* 0.05) (Table 2).

**Table 1:** Comparison of general information between patients with IBD and HCs.

Variable	HC (*n* = 47)	IBD (*n* = 96)	*p* value
Male	18 (38.30)	52 (54.17)	0.05
Age	28.57 ± 7.29	30.73 ± 8.11	0.13
BMI	21.15 ± 2.57	20.08 ± 3.20	**0.03**
Mean-VAS	0.00 (0.00, 0.00)	1 (0.00, 3.75)	**<0.001**
Max-VAS	0.00 (0.00, 0.00)	1 (0.00, 4.88)	**<0.001**
GIQLI	128.45 ± 11.65	107.00 ± 24.15	**<0.001**
FPQ	78.60 ± 18.57	76.50 ± 19.91	0.55
PASS	37.74 ± 15.11	39.52 ± 17.87	0.56
PCS	14.49 ± 8.07	16.04 ± 10.76	0.34
PSQ	2.38 ± 0.65	2.73 ± 1.33	**0.04**
PVAQ	36.74 ± 8.91	38.83 ± 14.32	0.29
BDI	5.70 ± 5.05	10.52 ± 8.53	**<0.001**
STAI	37.17 ± 8.27	41.20 ± 9.05	**0.01**

BMI, body mass index; Mean-VAS, mean visual analog scale; Max-VAS, max visual analog scale; GIQLI, gastrointestinal quality of life index; FPQ, fear of pain questionnaire; PASS, pain anxiety symptoms scale; PCS, pain catastrophizing scale; PSQ, pain sensitivity questionnaire; PVAQ, pain and vigilance awareness questionnaire; BDI, Beck Depression Inventory; STAI, State–Trait Anxiety Inventory. Bold values indicate statistically significant differences (*p* < 0.05).

**Table 2:** Comparison between CD and UC subtypes: demographic, clinical, and psychometric profiles.

Variable	CD (*n* = 64)	UC (*n* = 32)	*p* value
Male	39 (60.94)	13 (40.63)	0.06
Age	29.22 ± 7.27	33.75 ± 8.94	**0.01**
Disease duration	5.34 ± 4.78	4.84 ± 3.16	0.59
Surgery	38 (59.38)	0 (0.00)	**<0.001**
BMI	19.62 ± 2.86	21.01 ± 3.68	0.07
Disease activity	24 (37.50)	8 (25.00)	**0.01**
Mean-VAS	0.00 (0.00, 3.45)	3.00 (0.00, 4.90)	0.14
Max-VAS	0.00 (0.00, 4.00)	3 0.00 (0, 5.83)	0.18
GIQLI	111.47 ± 22.88	97.91 ± 24.44	**0.01**
FPQ	75.23 ± 21.19	79.03 ± 17.10	0.38
PASS	40.61 ± 17.98	37.34 ± 17.73	0.40
PCS	16.05 ± 10.86	16.03 ± 10.72	0.10
PSQ	2.66 ± 1.29	2.87 ± 1.40	0.45
PVAQ	37.61 ± 13.45	41.28 ± 15.87	0.24
BDI	9.86 ± 8.23	11.84 ± 9.09	0.29
STAI	40.83 ± 8.89	41.94 ± 9.48	0.57

BMI, body mass index; Mean-VAS, mean visual analog scale; Max-VAS, max visual analog scale; GIQLI, gastrointestinal quality of life index; FPQ, fear of pain questionnaire; PASS, pain anxiety symptoms scale; PCS, pain catastrophizing scale; PSQ, pain sensitivity questionnaire; PVAQ, pain and vigilance awareness questionnaire; BDI, Beck Depression Inventory; STAI, State–Trait Anxiety Inventory. Bold values indicate statistically significant differences (*p* < 0.05).

**Figure 1:**
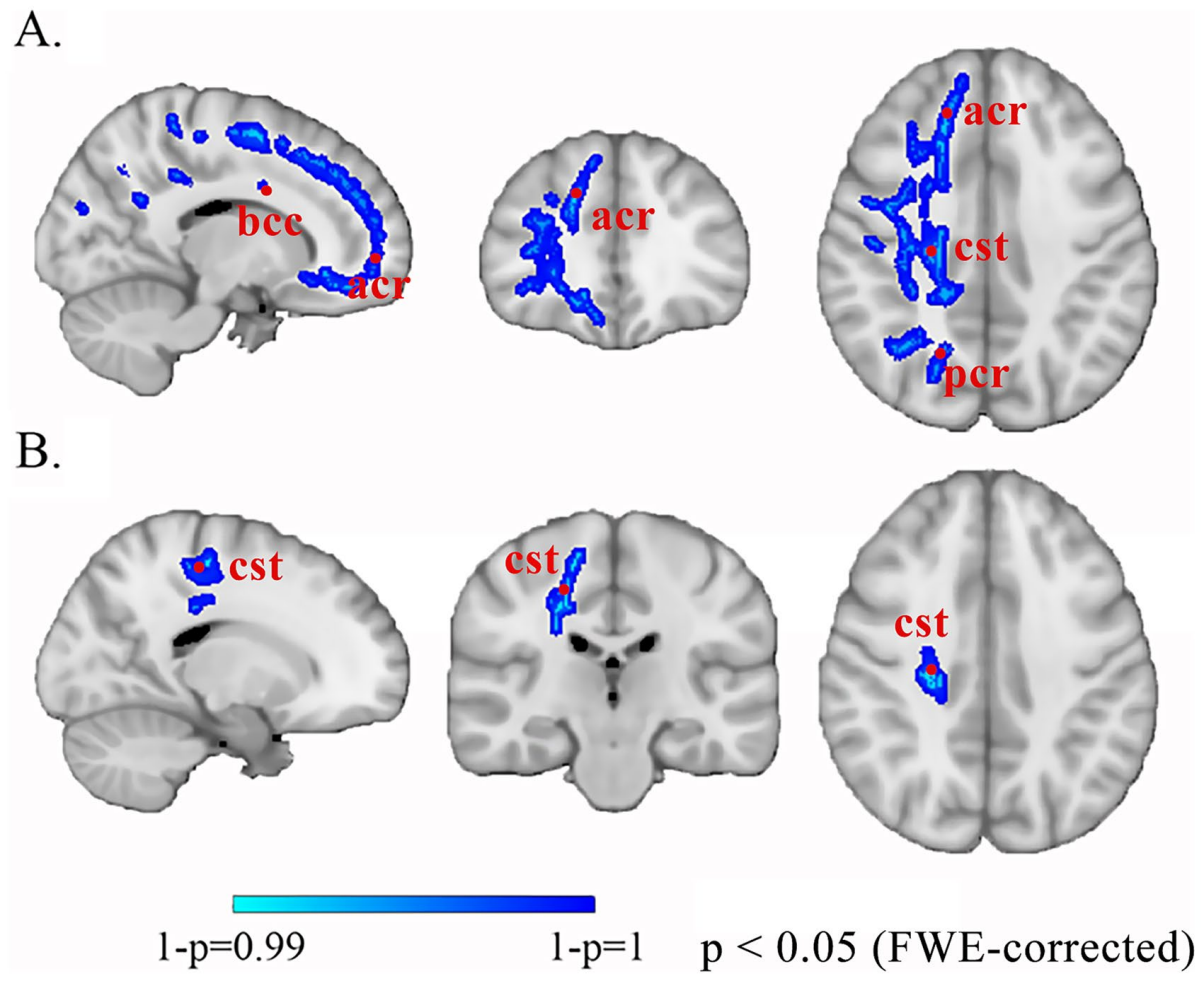
Altered white matter in patients with IBD compared with HCs. The blue-cyan scale represents negative significance, where color saturation scales with 1 − *p* values (deeper blues reflect 1 − *p* → 1, demonstrating higher confidence in decreased diffusion metrics after FWE correction). **(A)** MD. **(B)** AD. bcc, body of corpus callosum; acr, anterior corona radiata; cst, corticospinal tract; pcr, posterior corona radiata; FWE, family-wise error.

Comparison of differences in brain white matter between patients with IBD and healthy controls Compared with HCs, patients with IBD presented decreased MD values in the right corticospinal tract, body of the corpus callosum, posterior limb of the internal capsule, and anterior and posterior corona radiata (*p* < 0.01 FWE corrected); decreased AD values in the right corticospinal tract (Figure 1); and decreased RD values in the genu and splenium of the corpus callosum, bilateral corticospinal tracts, and left cingulum bundle (*p* < 0.05). No significant group differences in AD were observed (*p* > 0.05) (Table 3).

**Table 3:** Significantly abnormal white matter microstructure in patients with IBD and HCs.

DTI metrics	Abnormal white matter microstructure	*p* value
MD	Right corticospinal tract, body of corpus callosum, posterior limb of internal capsule, anterior and posterior corona radiata	**<0.01**
AD	Right corticospinal tract	**<0.01**
RD	Genu and splenium of the corpus callosum, bilateral corticospinal tracts, left cingulum bundle	**<0.05**
FA	/	>0.05

Bold values indicate statistically significant differences (*p* < 0.05).

**Figure 2:**
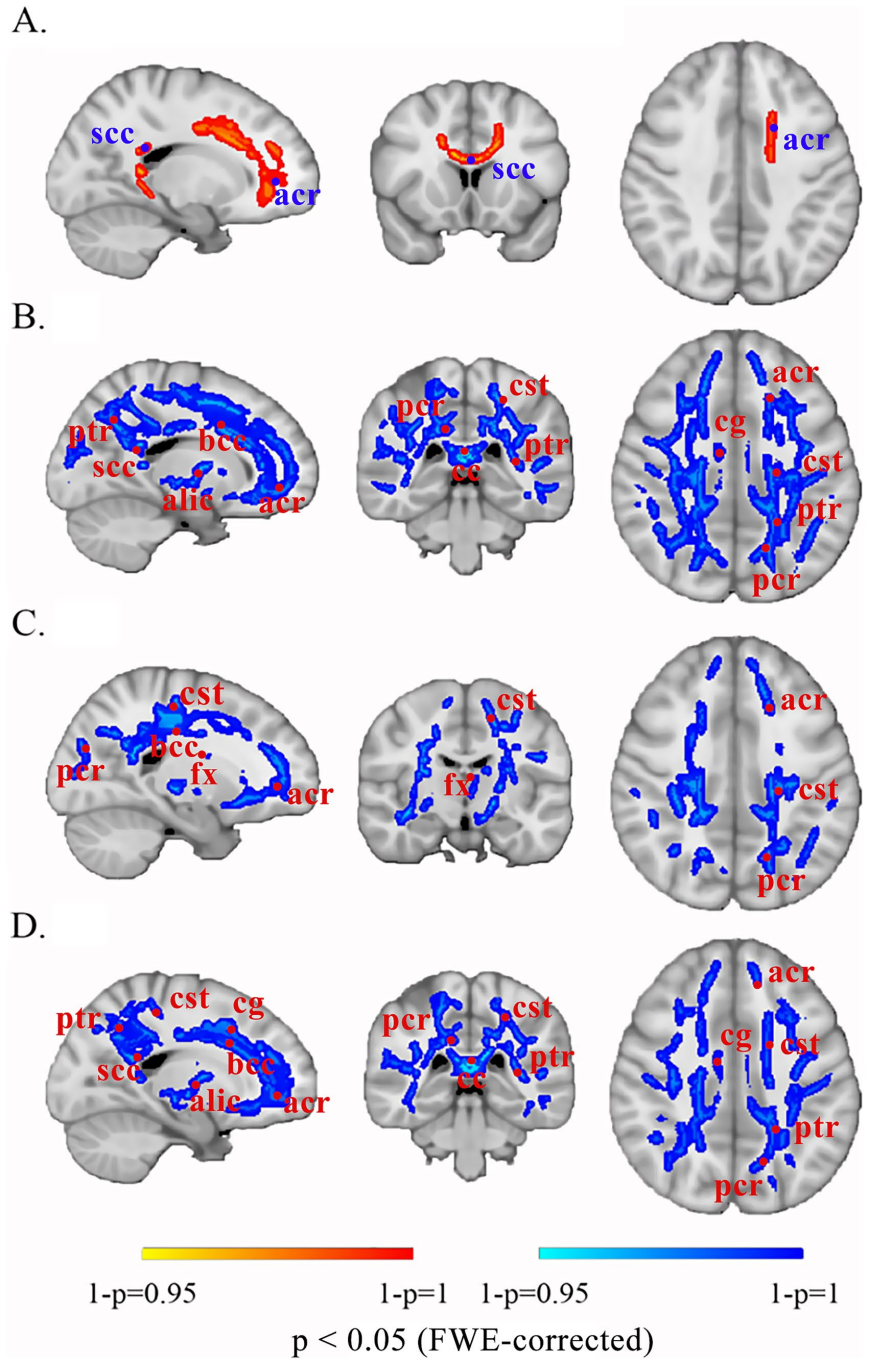
Altered white matter in patients with CD compared with that in the brains of HCs. The red-yellow scale denotes positive significance, with saturation intensity proportional to 1 − *p* values (darker hues correspond to 1 − *p* → 1, indicating greater statistical reliability of FA increases following FWE correction). The blue-cyan scale represents negative significance, with saturation intensity proportional to 1 − *p* values (deeper blues reflect 1 − *p* → 1, demonstrating higher confidence in decreased diffusion metrics after FWE correction). **(A)** FA. **(B)** MD. **(C)** AD. **(D)** RD. scc, splenium of the corpus callosum; acr, anterior corona radiata; ptr, posterior thalamic radiation; bcc, body of the corpus callosum; alic, anterior limb of the internal capsule; pcr, posterior corona radiata; cst, corticospinal tract; cc, corpus callosum; cg, cingulum; fx, fornix; FWE, family-wise error.

Compared with HCs, patients with UC presented no significant WM differences (all *p* > 0.05). Consequently, subsequent analyses focused on CD-specific alterations. Patients with CD displayed (1) increased FA in the splenium of the corpus callosum and left anterior corona radiata; (2) decreased MD/AD/RD in the body of the corpus callosum, bilateral corticospinal tracts, bilateral anterior and posterior corona radiata, and fornix; and (3) decreased MD/RD in the splenium of the corpus callosum, bilateral cingulum bundle, bilateral posterior limbs of the internal capsule, left anterior limb of the internal capsule, and left posterior thalamic radiation (*p* < 0.05) (Figure 2; Table 4).

**Table 4:** Significantly abnormal white matter microstructure in patients with CD and HCs.

DTI metrics	Abnormal white matter microstructure	*p* value
FA	Splenium of the corpus callosum, left anterior corona radiata	**<0.05**
MD	Bilateral corticospinal tracts, bilateral anterior and posterior corona radiata, body and splenium of corpus callosum, bilateral cingulum bundle, bilateral posterior limbs of internal capsule, left anterior limb of the internal capsule, left posterior thalamic radiation, fornix	**<0.05**
AD	Body of corpus callosum, bilateral corticospinal tracts, bilateral anterior and posterior corona radiata, fornix	**<0.05**
RD	Bilateral corticospinal tracts, bilateral anterior and posterior corona radiata, body and splenium of corpus callosum, bilateral cingulum bundle, bilateral posterior limbs of internal capsule, left anterior limb of the internal capsule, left posterior thalamic radiation, fornix	**<0.05**

Bold values indicate statistically significant differences (*p* < 0.05).

### 3.2 PLSC analysis between behavioral and whole-brain white matter microstructure imaging

**Figure 3:**
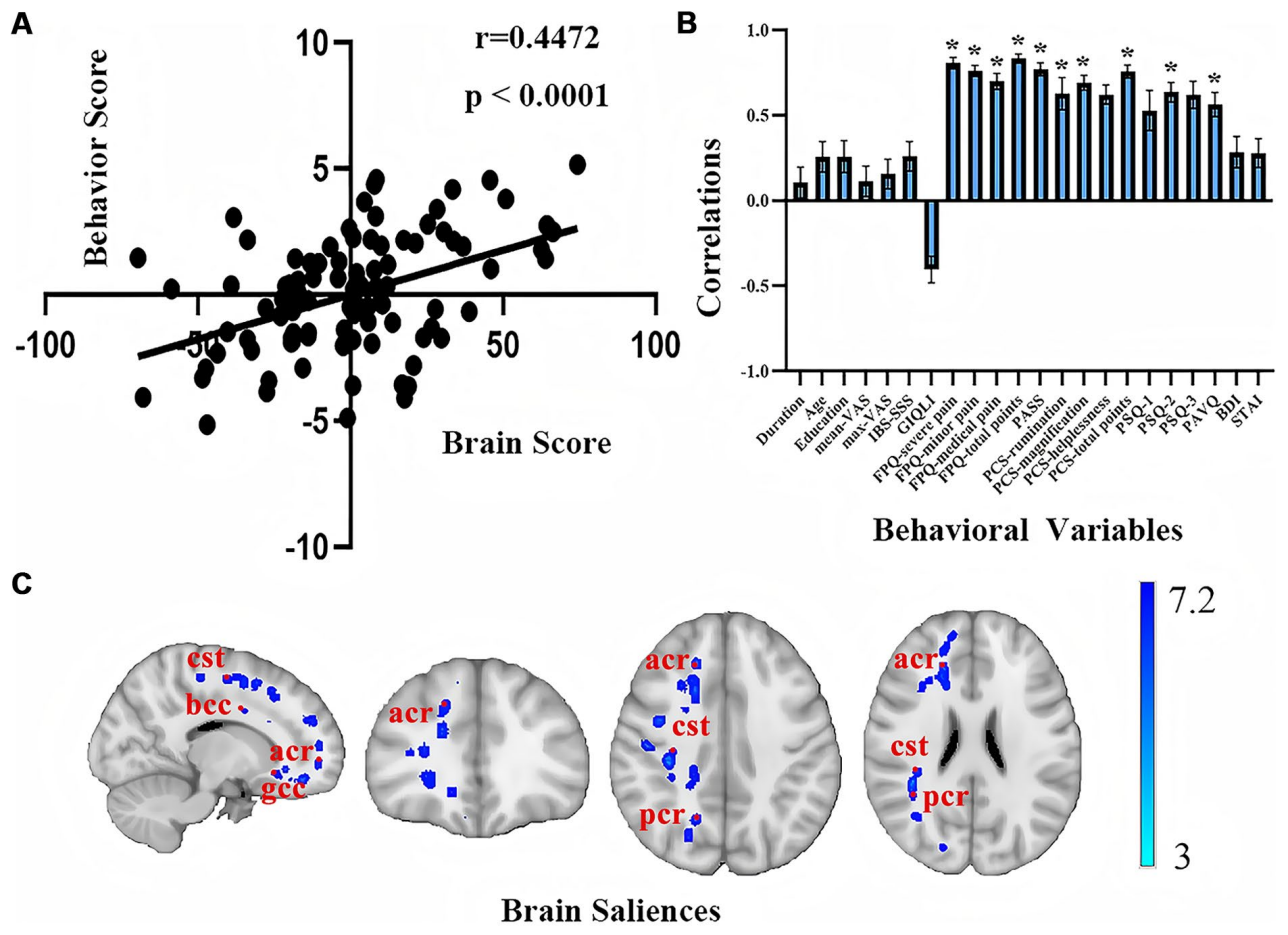
PLSC analysis results of behavioral and white matter structural imaging in patients with IBD. **(A)** Scatter plot of the behavioral traits correlated with the WM microstructure; **(B)** Coefficient plot of the behavioral traits correlated with the WM of the brain; **(C)** Significant brain regions of the WM in the PLSC analysis. The blue-cyan scale represents negative significance, with saturation intensity proportional to 1 − *p* values (deeper blues reflect 1 − *p* → 1, demonstrating higher confidence in decreased diffusion metrics after FWE correction). bcc, body of corpus callosum; cst, corticospinal tract; acr, anterior corona radiata; gcc, genu of the corpus callosum; pcr, posterior corona radiata.

In the PLSC analysis of behavior and WM in all patients with IBD, the first latent variable (LV1) was a significant main result (*r* = 0.4472, *p* < 0.0001), explaining 35% of the correlation between behavior and imaging (Figure 3). The negative emotions related to pain in patients, including the FPQ, PASS, PCS, PSQ, and PVAQ, demonstrated significant positive correlations (*p* < 0.05). The duration of illness, age, level of education, VAS score, BDI score, STAI score, and severity of IBD showed nonsignificant positive relationships; whereas the GIQLI score exhibited a nonsignificant negative relationship (*p* > 0.05). As shown in Figure 3C, the significant brain matrix of LV1 in the WM microstructure PLSC was mapped onto the standard brain template image, obtaining images of significant brain regions on the WM skeleton. The significant negative brain regions in patients with IBD were distributed mainly in the right anterior and posterior corona radiata, right corticospinal tract, body, and genu of the corpus callosum.

## 4 Discussion

In this study, patients with IBD, especially those with CD, had WM changes in multiple regions, including the corticospinal tract, corona radiata, and corpus callosum, when compared with HCs. These changes suggest that the integrity of the WM fiber tracts in these patients is compromised. Brain WM consists of bundles of axons that connect different regions of the brain and serve as carriers for information transmission during functional brain activities. Therefore, damage to WM may affect the transmission of neurological information, which in turn affects brain function and further contributes to neurological disorders (Zhao et al., 2021). In addition, the multivariate analysis revealed that structural changes in WM were positively correlated with pain-related negative emotion scores (such as the FPQ, PASS, and PCS), suggesting that microstructural changes in WM in patients with IBD may be closely related to pain perception and emotional regulation. This association may be linked to a proposed mechanism that persistent or recurrent pain signals in patients with IBD ascend through vagal afferent fibers and the spinothalamic pathway, activating central sensitization mechanisms and promoting plastic remodeling of brain structures (Latremoliere and Woolf, 2009). Concurrently, circulating inflammatory mediators (such as TNF-α and IL-6) disrupt the blood–brain barrier, activate microglia and astrocytes in the brain, and induce oligodendrocyte apoptosis and excitotoxicity through the release of proinflammatory factors, further contributing to WM microstructural changes. Such structural remodeling of the brain may further increase pain sensitivity and amplify pain perception through emotion–pain interactions, establishing a vicious cycle of pain–neural remodeling–emotional dysfunction (Latremoliere and Woolf, 2009).

Compared with HCs, patients with CD presented increased FA values in the splenium of the corpus callosum and the left anterior corona radiata, alongside decreased MD, AD, and RD values in the corticospinal tract, corona radiata, and corpus callosum. FA quantifies the directionality of water molecule diffusion along WM tracts, serving as a key indicator of microstructural integrity (Leng et al., 2018). Its elevation reflects improved fiber alignment coherence and myelination maturity (Montaser-Kouhsari et al., 2022; Stewart et al., 2023). Previous studies have suggested that increased FA may be associated with neuroplastic changes. For example, Tan et al. (2022) demonstrated that elevated FA in pain-processing regions coincided with increased gray matter density or myelinated axon density, indicating an adaptive response to chronic pathological stimuli. In terms of the diffusion metrics, RD and AD reflect the diffusion rates of water molecules perpendicular and parallel to the axons, respectively, (Xi et al., 2016), whereas MD represents the average diffusion rate in all directions (Maleki et al., 2020). Notably, diffusivity metrics (RD/AD/MD) demonstrate high sensitivity to microstructural disruption (axonal injury and demyelination), and their reduction may indicate occult WM pathology (Liu et al., 2012). Therefore, the observed parameter constellation in this study (increased FA with decreased MD, AD, and RD) suggests compromised neural signal transmission in these regions, potentially associated with microstructural damage or chronic adaptive reorganization of WM. The corpus callosum, which connects the left and right brain hemispheres, plays a key role in interhemispheric communication, particularly the frontal lobe (Roland et al., 2017). Abnormalities in its microstructure could lead to emotional imbalances, favoring negative emotions due to ineffective inhibition of the right frontal lobe by the left frontal lobe. The corona radiata is a radiating WM fiber bundle located between the internal capsule and the cerebral cortex, forming part of the frontal lobe to limbic circuitry and playing an important role in emotion regulation and sensory conduction (Nakajima et al., 2020). One animal study (Liu et al., 2018) showed that certain corticospinal neurons originating in the primary and secondary somatosensory cortex can modulate touch-induced pain by connecting the spinal dorsal horn. Moreover, a recent study (Fan et al., 2024) demonstrated that the establishment and maintenance of pain perception in central poststroke pain depends on the activation of postsynaptic spinal cord targets of corticospinal neurons. In the present study, the, multivariate analysis results indicate that WM changes in the corticospinal tract, corona radiata, and corpus callosum in patients with IBD are positively correlated with pain-related negative emotions, underscoring their potential impact on pain perception and emotional regulation.

In addition, in the PLSC analysis of disease behavior and WM structure in this study, no statistically significant associations were found between WM changes in patients with IBD and anxiety or depression, though a positive correlation trend was still observed. This phenomenon is highly consistent with previous studies regarding mental comorbidities in patients with IBD, where the prevalence of anxiety and depression in patients with IBD (especially those with CD) is significantly greater than that in healthy individuals, and the severity of emotional disorders is positively correlated with disease activity (Barberio et al., 2021; Mikocka-Walus et al., 2016). From a neurobiological perspective, this association may be closely related to chronic abdominal pain in IBD and the remodeling of WM microstructure. Neurofiber injury models have demonstrated that pain can induce anxiety-like behavior in mice, suggesting that neural pathways are shared between pain transmission and emotional regulation (Yamauchi et al., 2022). In contrast, recent animal studies have revealed that specific neural circuits in the anterior paraventricular thalamic nucleus (aPVT) and posterior paraventricular thalamic nucleus (pPVT) mediate visceral pain and anxiety responses, respectively (Li et al., 2025). Notably, the WM alteration regions in patients with IBD (such as the corona radiata, corpus callosum, and posterior thalamic radiation) precisely overlap with key connection nodes of these circuits, suggesting that impaired neural transmission efficiency in the pain–emotion integration network may indirectly promote the onset and progression of psychiatric symptoms.

However, in contrast to the results of the present study, a previous study (Prüß et al., 2022) found no significant abnormalities in brain WM in patients with IBD with chronic abdominal pain compared with HCs. This discrepancy may be attributed to the unclear ratio of the two IBD phenotypes (CD and UC) included in their study, as well as differences in the disease states of the patients. In the present study, no significant differences between patients with UC and HCs were observed, whereas patients with CD presented abnormalities in multiple regions of WM tracts. These findings suggest that the significant differences in brain WM between patients with IBD and HCs are largely attributable to CD. This phenomenon may be related to the higher previous surgery rates and greater disease activity observed in the current cohort of patients with CD. In addition, the findings of the present study are consistent with those of a systematic review regarding psychiatric disorders in patients with IBD, which indicated that patients with CD have a greater prevalence of psychiatric disorders than those with UC (Arp et al., 2022). This difference may reflect the complexity of disease progression and the neurobiological mechanisms involved in the two IBD phenotypes. CD is characterized by chronic transmural segmental inflammation involving the entire intestinal wall (Dolinger et al., 2024), disrupts the intestinal mucosal barrier, increases permeability and facilitates bacterial translocation (such as *C. innocuum*) to mesenteric adipose tissue. This process stimulates hyperplasia of mesenteric fat, resulting in the formation of CD-specific creeping fat (Ha et al., 2020). Creeping fat serves as a significant source of proinflammatory cytokines (e.g., IL-6 and TNF-α), which exacerbate local and systemic inflammation through NF-κB pathway activation (Takahashi et al., 2017). Furthermore, studies indicate that CD pathogenesis involves the enteric nervous system, where mesenteric nerves—critical components of the gut–brain axis—mediate physiological communication between the central nervous system and gastrointestinal tract (Villanacci et al., 2008). Transmural inflammation directly damages vagal nerve terminals within the intestinal wall and mesenteric plexuses, disrupting gut–brain axis signaling through the inhibition of the α7 nicotinic acetylcholine receptor (α7nAChR)-mediated cholinergic anti-inflammatory pathway (Di Giovangiulio et al., 2016). In contrast, UC primarily affects the colorectum with inflammation confined to the mucosa and submucosa (Le Berre et al., 2023), exerting minimal effects on deep neural structures and systemic immunity. Consequently, the specific alterations in WM structure observed in patients with CD may be due to a multidimensional pathological cascade initiated by transmural inflammation: from barrier disruption-induced bacterial translocation and systemic inflammation to mesenteric neural injury-mediated disruption of gut–brain axis signaling, ultimately driving structural remodeling within the central nervous system. These findings not only substantiate fundamental neuropathological distinctions between CD and UC but also provide critical insights for developing novel therapeutic strategies targeting the gut–brain axis in CD.

The results of the present study suggest that IBD involves not only intestinal symptoms but also complex bidirectional interactions with the central nervous system via the brain–gut axis. In patients with IBD, alterations in WM appear to be correlated with gastrointestinal symptoms mediated through gut-to-brain signaling pathways, clinically manifesting as increased pain sensitivity and reduced pain thresholds. Conversely, persistent chronic abdominal pain may correlate with adaptive neuroplastic changes in a reciprocal manner (Turkiewicz et al., 2021). The brain–gut axis is a bidirectional communication pathway consisting of the autonomic nervous system, neuroendocrine pathways, and gastrointestinal tract (Chakrabarti et al., 2022; Mogilevski et al., 2019; Qiu et al., 2022). Among them, the autonomic nervous system, particularly the vagus nerve, has been shown to regulate neural reflexes in the gut and influence the onset and progression of IBD (Mikami et al., 2022). An intact vagus nerve can exert anti-inflammatory effects by releasing acetylcholine (Mikami et al., 2022). However, dysregulation of the autonomic nervous system can compromise the integrity of the gastrointestinal mucosal barrier (Singh et al., 2016). Additionally, intestinal ischemia–reperfusion can lead to excessive production of nitrates, resulting in elevated levels of Enterobacteriaceae and ultimately causing dysbiosis of the gut microbiota (Xu et al., 2021). Metabolites of the gut microbiota, such as short-chain fatty acids, gamma-aminobutyric acid, and glutamate, can be utilized in the brain via the gut–brain axis (Gilca-Blanariu et al., 2023; Guo et al., 2022) to regulate host behavior, mood, and cognition (Tang et al., 2023). For example, in patients with CD suffering from abdominal pain, there are significant changes in the metabolite status of the bilateral anterior cingulate cortex, which plays a key role in pain processing in patients with CD (Kong et al., 2022). Collectively, these findings indicate that early implementation of gut–brain axis-based anti-inflammatory interventions in patients with IBD represents a promising translational strategy achievable through multiple approaches. At the neuromodulatory level, vagus nerve stimulation may facilitate clinical, biochemical, and endoscopic remission (Bonaz et al., 2016; D'Haens et al., 2023) via acetylcholine-mediated activation of the α7nAChR pathway to suppress TNF-α release (Sundman and Olofsson, 2014). For microbial modulation, fecal microbiota transplantation corrects dysbiosis and restores beneficial microbiota, with two randomized controlled trials regarding CD demonstrating its efficacy in inducing and maintaining clinical remission (Sokol et al., 2020; Yang et al., 2020). Pharmacological agents targeting neurotransmitter receptors also have therapeutic importance, as low-dose antidepressants ameliorate intestinal inflammation by reducing mesenteric afferent nerve activity (Yin et al., 2021). In summary, early intervention targeting the gut–brain axis not only has the potential to overcome conventional therapeutic limitations but also may enable critical modulation of disease pathogenesis. Future research must prioritize elucidating intricate gut–brain interaction mechanisms, identifying key regulatory nodes, and developing personalized strategies to shift IBD management from symptomatic control to a mechanism-targeted cure.

This study has several limitations. First, the restricted sample size precluded subgroup analyses on the disease activity and remission status. Future research should expand cohorts to comprehensively characterize WM microstructural changes across different disease states. Second, the participants were not stratified by their duration of abdominal pain. Subsequent studies may conduct gradient analyses across defined pain chronicity categories—specifically, recent abdominal pain (onset ≤ 4 weeks), intermittent abdominal pain within 1 year (≥3 recurrent episodes within 1 year with symptom-free intervals) and persistent abdominal pain (continuous pain ≥3 months)—to more precisely elucidate the dynamic effects of pain chronicity on the central nervous system. Third, as a cross-sectional design, this study inherently precludes causal inferences regarding abdominal pain and WM alterations. Future longitudinal studies tracking WM dynamics along pain progression, coupled with targeted interventions (such as vagus nerve stimulation), are needed to establish temporal relationships and causality. Finally, the findings may be influenced by confounders (including the use of immunosuppressants or antidepressants) and limited generalizability due to recruitment from a single center. Regional factors such as dietary habits or genetic backgrounds specific to this cohort may restrict the extrapolation of results to broader IBD populations. Future research must incorporate multivariate analyses to control for pharmacological confounders and employ multicenter recruitment strategies to increase external validity.

## 5 Conclusions

In this study, we investigated the differences in WM microstructure between patients with IBD and HCs using DTI. Through PLSC analysis, an effective method for identifying multivariate relationships, WM alterations in patients were found to be significantly correlated with negative emotional perceptions, particularly pain-related manifestations. Chronic abdominal pain, a prevalent symptom of IBD, substantially compromises quality of life and emotional well-being. Critically, within the gut–brain axis framework, persistent abdominal pain and brain WM changes exhibit bidirectional interdependence, wherein neuroadaptive processes may intensify pain perception while nociceptive signaling potentially facilitates microstructural remodeling. These reciprocal interactions can impair patients’ disease management abilities, reduce treatment adherence, and ultimately influence clinical outcomes (Hasan and Nicolaidis, 2020). Therefore, in the treatment and management of patients with IBD, in addition to addressing the relief of intestinal symptoms, attention should also be given to negative emotional issues related to brain microstructure and function. Furthermore, effectively utilizing the therapeutic potential may have important implications for the future development of IBD treatment strategies.

## Data Availability

The raw data supporting the conclusions of this article will be made available by the authors, without undue reservation.

## References

[ref1] Agostini A. Benuzzi F. Ballotta D. Rizzello F. Gionchetti P. Filippini N. (2023). Differential brain structural and functional patterns in crohn's disease patients are associated with different disease stages. Inflamm. Bowel Dis. 29, 1297–1305. doi: 10.1093/ibd/izad029, PMID: 36897213

[ref2] Andus T. Roth M. Kullmann F. Caesar I. Gross V. Feuerbach S.. (1995). Focal white-matter lesions in brain of patients with inflammatory bowel disease. Lancet 345, 897–898. doi: 10.1016/S0140-6736(95)90013-6, PMID: 7707814

[ref3] Arp L. Jansson S. Wewer V. Burisch J. (2022). Psychiatric disorders in adult and paediatric patients with inflammatory bowel diseases – a systematic review and Meta-Analysis. J. Crohn's Colitis 16, 1933–1945. doi: 10.1093/ecco-jcc/jjac095, PMID: 35775920

[ref4] Barberio B. Zamani M. Black C. J. Savarino E. V. Ford A. C. (2021). Prevalence of symptoms of anxiety and depression in patients with inflammatory bowel disease: A systematic review and meta-analysis. Lancet Gastroenterol. Hepatol. 6, 359–370. doi: 10.1016/S2468-1253(21)00014-5, PMID: 33721557

[ref5] Bonaz B. Sinniger V. Hoffmann D. Clarencon D. Mathieu N. Dantzer C.. (2016). Chronic vagus nerve stimulation in Crohn's disease: A 6-month follow-up pilot study. Neurogastroenterol. Motil. 28, 948–953. doi: 10.1111/nmo.12792, PMID: 26920654

[ref6] Chakrabarti A. Geurts L. Hoyles L. Iozzo P. Kraneveld A. D. La Fata G.. (2022). The microbiota–gut–brain axis: Pathways to better brain health. Perspectives on what we know, what we need to investigate and how to put knowledge into practice. Cell. Mol. Life Sci. 79:80. doi: 10.1007/s00018-021-04060-w, PMID: 35044528 PMC8770392

[ref7] Chang J. T. (2020). Pathophysiology of inflammatory bowel diseases. N. Engl. J. Med. 383, 2652–2664. doi: 10.1056/NEJMra2002697, PMID: 33382932

[ref8] Chen M. Lee G. Kwong L. N. Lamont S. Chaves C. (2012). Cerebral white matter lesions in patients with crohn's disease. J. Neuroimaging 22, 38–41. doi: 10.1111/j.1552-6569.2010.00538.x, PMID: 21091817

[ref9] Croall I. D. Sanders D. S. Hadjivassiliou M. Hoggard N. (2020). Cognitive deficit and white matter changes in persons with celiac disease: A Population-Based study. Gastroenterology 158, 2112–2122. doi: 10.1053/j.gastro.2020.02.028, PMID: 32088203

[ref10] D'Haens G. Eberhardson M. Cabrijan Z. Danese S. van den Berg R. Lowenberg M.. (2023). Neuroimmune modulation through vagus nerve stimulation reduces inflammatory activity in Crohn's disease patients: a prospective open-label study. J. Crohns Colitis 17, 1897–1909. doi: 10.1093/ecco-jcc/jjad151, PMID: 37738465 PMC10798868

[ref11] Di Giovangiulio M. Bosmans G. Meroni E. Stakenborg N. Florens M. Farro G.. (2016). Vagotomy affects the development of oral tolerance and increases susceptibility to develop colitis independently of the alpha-7 nicotinic receptor. Mol. Med. 22, 464–476. doi: 10.2119/molmed.2016.00062, PMID: 27341335 PMC5072409

[ref7001] Dolinger M. Torres J. Vermeire S. (2024). Crohn’s disease. The Lancet. 403, 1177–1191. doi: 10.1016/S0140-6736(23)02586-2 38437854

[ref12] Fan F. Yin T. Wu B. Zheng J. Deng J. Wu G.. (2024). The role of spinal neurons targeted by corticospinal neurons in central poststroke neuropathic pain. CNS Neurosci. Ther. 30:e14813. doi: 10.1111/cns.14813, PMID: 38887838 PMC11183184

[ref13] Gilca-Blanariu G. E. Schiopu C. G. Stefanescu G. Mihai C. Diaconescu S. Afrasanie V. A.. (2023). The intertwining roads between psychological distress and gut microbiota in inflammatory bowel disease. Microorganisms 11:2268. doi: 10.3390/microorganisms11092268, PMID: 37764111 PMC10538137

[ref14] Guo G. Tan Z. Liu Y. Shi F. She J. (2022). The therapeutic potential of stem cell-derived exosomes in the ulcerative colitis and colorectal cancer. Stem Cell Res Ther 13:138. doi: 10.1186/s13287-022-02811-5, PMID: 35365226 PMC8973885

[ref15] Ha C. Martin A. Sepich-Poore G. D. Shi B. Wang Y. Gouin K.. (2020). Translocation of viable gut microbiota to mesenteric adipose drives formation of creeping fat in humans. Cell 183, 666–683. doi: 10.1016/j.cell.2020.09.009 32991841 PMC7521382

[ref16] Hasan R. Nicolaidis C. (2020). Psychiatric comorbidities increase cost and length of hospitalization in adolescents and young adults with chronic medical conditions. J. Gen. Intern. Med. 35, 1897–1899. doi: 10.1007/s11606-020-05726-0, PMID: 32076983 PMC7280366

[ref17] Hou J. Dodd K. Nair V. A. Rajan S. Beniwal-Patel P. Saha S.. (2020). Alterations in brain white matter microstructural properties in patients with Crohn's disease in remission. Sci. Rep. 10:2145. doi: 10.1038/s41598-020-59098-w, PMID: 32034257 PMC7005825

[ref18] Hung C. Chao Y. Lee Y. Huang C. Huang S. Chang C.. (2023). Cingulate white matter mediates the effects of fecal *Ruminococcus* on neuropsychiatric symptoms in patients with amyloid-positive amnestic mild cognitive impairment. BMC Geriatr. 23:720. doi: 10.1186/s12877-023-04417-9, PMID: 37936084 PMC10631051

[ref19] Kong N. Gao C. Zhang F. Zhang M. Yue J. Lv K.. (2022). Neurophysiological effects of the anterior cingulate cortex on the exacerbation of crohn's disease: A combined fMRI-MRS study. Front. Neurosci. 16:840149. doi: 10.3389/fnins.2022.840149, PMID: 35600612 PMC9120361

[ref20] Latremoliere A. Woolf C. J. (2009). Central sensitization: A generator of pain hypersensitivity by central neural plasticity. J. Pain 10, 895–926. doi: 10.1016/j.jpain.2009.06.012, PMID: 19712899 PMC2750819

[ref21] Le Berre C. Honap S. Peyrin-Biroulet L. (2023). Ulcerative colitis. Lancet 402, 571–584. doi: 10.1016/S0140-6736(23)00966-2, PMID: 37573077

[ref22] Leng X. Lan L. Ip H. L. Fan F. Ma S. H. Ma K.. (2018). Translesional pressure gradient and leptomeningeal collateral status in symptomatic middle cerebral artery stenosis. Eur. J. Neurol. 25, 404–410. doi: 10.1111/ene.13521, PMID: 29171118

[ref23] Li D. Li Y. C. Zhu Z. Y. Zhang F. C. Zhao Q. Y. Jiang J. H.. (2025). The paraventricular thalamus mediates visceral pain and anxiety-like behaviors via two distinct pathways. Neuron. doi: 10.1016/j.neuron.2025.04.019, PMID: 40345185

[ref24] Liu Y. Duan Y. He Y. Yu C. Wang J. Huang J.. (2012). A tract-based diffusion study of cerebral white matter in neuromyelitis optica reveals widespread pathological alterations. Mult. Scler. 18, 1013–1021. doi: 10.1177/1352458511431731, PMID: 22183932

[ref25] Liu Y. Latremoliere A. Li X. Zhang Z. Chen M. Wang X.. (2018). Touch and tactile neuropathic pain sensitivity are set by corticospinal projections. Nature (London) 561, 547–550. doi: 10.1038/s41586-018-0515-2, PMID: 30209395 PMC6163083

[ref26] Maleki S. Chye Y. Zhang X. Parkes L. Chamberlain S. R. Fontenelle L. F.. (2020). Neural correlates of symptom severity in obsessive-compulsive disorder using magnetization transfer and diffusion tensor imaging. Psychiatry Res. Neuroimaging 298:111046. doi: 10.1016/j.pscychresns.2020.111046, PMID: 32106018 PMC7100004

[ref27] Mikami Y. Tsunoda J. Kiyohara H. Taniki N. Teratani T. Kanai T. (2022). Vagus nerve-mediated intestinal immune regulation: Therapeutic implications of inflammatory bowel diseases. Int. Immunol. 34, 97–106. doi: 10.1093/intimm/dxab039, PMID: 34240133

[ref28] Mikocka-Walus A. Knowles S. R. Keefer L. Graff L. (2016). Controversies revisited: a systematic review of the comorbidity of depression and anxiety with inflammatory bowel diseases. Inflamm. Bowel Dis. 22, 752–762. doi: 10.1097/MIB.0000000000000620, PMID: 26841224

[ref29] Mogilevski T. Burgell R. Aziz Q. Gibson P. R. (2019). Review article: the role of the autonomic nervous system in the pathogenesis and therapy of IBD. Aliment. Pharmacol. Ther. 50, 720–737. doi: 10.1111/apt.15433, PMID: 31418887

[ref30] Montaser-Kouhsari L. Young C. B. Poston K. L. (2022). Neuroimaging approaches to cognition in Parkinson's disease. Prog. Brain Res. 269, 257–286. doi: 10.1016/bs.pbr.2022.01.008, PMID: 35248197

[ref31] Mowat C. Cole A. Windsor A. Ahmad T. Arnott I. Driscoll R.. (2011). Guidelines for the management of inflammatory bowel disease in adults. Gut 60, 571–607. doi: 10.1136/gut.2010.224154, PMID: 21464096

[ref32] Nakajima R. Kinoshita M. Shinohara H. Nakada M. (2020). The superior longitudinal fascicle: Reconsidering the fronto-parietal neural network based on anatomy and function. Brain Imaging Behav. 14, 2817–2830. doi: 10.1007/s11682-019-00187-4, PMID: 31468374

[ref33] Prüß M. S. Bayer A. Bayer K. Schumann M. Atreya R. Mekle R.. (2022). Functional brain changes due to chronic abdominal pain in inflammatory bowel disease: a case-control magnetic resonance imaging study. Clin. Transl. Gastroenterol. 13:e453. doi: 10.14309/ctg.0000000000000453 PMC8865502 35060939

[ref34] Qiu Y. Li Q. Wu D. Zhang Y. Cheng J. Cao Z.. (2022). Altered mean apparent propagator-based microstructure and the corresponding functional connectivity of the parahippocampus and thalamus in Crohn's disease. Front. Neurosci. 16:985190. doi: 10.3389/fnins.2022.985190, PMID: 36203806 PMC9530355

[ref35] Roland J. L. Snyder A. Z. Hacker C. D. Mitra A. Shimony J. S. Limbrick D. D.. (2017). On the role of the corpus callosum in interhemispheric functional connectivity in humans. Proc. Natl. Acad. Sci. USA 114, 13278–13283. doi: 10.1073/pnas.1707050114, PMID: 29183973 PMC5740665

[ref36] Singh V. Roth S. Llovera G. Sadler R. Garzetti D. Stecher B.. (2016). Microbiota Dysbiosis Controls the Neuroinflammatory Response after Stroke. J. Neurosci. 36, 7428–7440. doi: 10.1523/JNEUROSCI.1114-16.2016, PMID: 27413153 PMC6705544

[ref37] Sokol H. Landman C. Seksik P. Berard L. Montil M. Nion-Larmurier I.. (2020). Fecal microbiota transplantation to maintain remission in Crohn's disease: a pilot randomized controlled study. Microbiome 8:12. doi: 10.1186/s40168-020-0792-5, PMID: 32014035 PMC6998149

[ref38] Stewart S. A. Pimer L. Fisk J. D. Rusak B. Leslie R. A. Eskes G.. (2023). Olfactory function and diffusion tensor imaging as markers of mild cognitive impairment in early stages of parkinson's disease. Clin. EEG Neurosci. 54, 91–97. doi: 10.1177/15500594211058263, PMID: 34841903 PMC9693894

[ref39] Sundman E. Olofsson P. S. (2014). Neural control of the immune system. Adv. Physiol. Educ. 38, 135–139. doi: 10.1152/advan.00094.2013, PMID: 25039084 PMC4056170

[ref40] Takahashi Y. Sato S. Kurashima Y. Lai C. Y. Otsu M. Hayashi M.. (2017). Reciprocal inflammatory signaling between intestinal epithelial cells and adipocytes in the absence of immune cells. EBioMedicine 23, 34–45. doi: 10.1016/j.ebiom.2017.07.027, PMID: 28789943 PMC5605307

[ref41] Tan Y. Zhou C. He L. (2022). Altered structural and functional abnormalities of hippocampus in classical trigeminal neuralgia: A combination of DTI and fMRI study. J. Healthc. Eng. 2022, 1–7. doi: 10.1155/2022/8538700, PMID: 36504636 PMC9729045

[ref42] Tang H. Chen X. Huang S. Yin G. Wang X. Shen G. (2023). Targeting the gut-microbiota-brain axis in irritable bowel disease to improve cognitive function - recent knowledge and emerging therapeutic opportunities. Rev. Neurosci. 34, 763–773. doi: 10.1515/revneuro-2022-0155, PMID: 36757367

[ref43] Thapaliya G. Eldeghaidy S. Asghar M. McGing J. Radford S. Francis S.. (2023). The relationship between Central Nervous System morphometry changes and key symptoms in Crohn’s disease. Brain Imaging Behav. 17, 149–160. doi: 10.1007/s11682-022-00742-6, PMID: 36409402 PMC10049962

[ref44] Thomann A. K. Schmitgen M. M. Kmuche D. Ebert M. P. Thomann P. A. Szabo K.. (2021). Exploring joint patterns of brain structure and function in inflammatory bowel diseases using multimodal data fusion. Neurogastroenterol. Motil. 33:e14078. doi: 10.1111/nmo.14078, PMID: 33368950

[ref45] Turkiewicz J. Bhatt R. R. Wang H. Vora P. Krause B. Sauk J. S.. (2021). Altered brain structural connectivity in patients with longstanding gut inflammation is correlated with psychological symptoms and disease duration. Neuroimage Clin. 30:102613. doi: 10.1016/j.nicl.2021.102613, PMID: 33823388 PMC8050027

[ref46] Villanacci V. Bassotti G. Nascimbeni R. Antonelli E. Cadei M. Fisogni S.. (2008). Enteric nervous system abnormalities in inflammatory bowel diseases. Neurogastroenterol. Motil. 20, 1009–1016. doi: 10.1111/j.1365-2982.2008.01146.x, PMID: 18492026

[ref47] Xi Y. B. Guo F. Li H. Chang X. Sun J. B. Zhu Y. Q.. (2016). The structural connectivity pathology of first-episode schizophrenia based on the cardinal symptom of auditory verbal hallucinations. Psychiatry Res. Neuroimaging 257, 25–30. doi: 10.1016/j.pscychresns.2016.09.011, PMID: 27744190

[ref48] Xu K. Gao X. Xia G. Chen M. Zeng N. Wang S.. (2021). Rapid gut dysbiosis induced by stroke exacerbates brain infarction in turn. Gut 70, 1486–1494. doi: 10.1136/gutjnl-2020-323263, PMID: 33558272

[ref49] Yamauchi N. Sato K. Sato K. Murakawa S. Hamasaki Y. Nomura H.. (2022). Chronic pain-induced neuronal plasticity in the bed nucleus of the stria terminalis causes maladaptive anxiety. Sci. Adv. 8:j5586. doi: 10.1126/sciadv.abj5586 PMC9045713 35476439

[ref50] Yang Z. Bu C. Yuan W. Shen Z. Quan Y. Wu S.. (2020). Fecal microbiota transplant via endoscopic delivering through small intestine and colon: No difference for crohn's disease. Dig. Dis. Sci. 65, 150–157. doi: 10.1007/s10620-019-05751-y, PMID: 31367877

[ref51] Yang C. Feng Z. Deng H. Dai L. He L. Yin L.. (2022). CXCL1/CXCR2 is involved in white matter injury in neonatal rats via the gut–brain axis. BMC Neurosci. 23:67. doi: 10.1186/s12868-022-00749-1, PMID: 36401162 PMC9675237

[ref52] Yin Y. Zhu Z. X. Li Z. Chen Y. S. Zhu W. M. (2021). Role of mesenteric component in Crohn's disease: A friend or foe? World J. Gastrointest. Surg. 13, 1536–1549. doi: 10.4240/wjgs.v13.i12.1536, PMID: 35070062 PMC8727179

[ref53] Zhao B. Li T. Yang Y. Wang X. Luo T. Shan Y.. (2021). Common genetic variation influencing human white matter microstructure. Science 372:eabf3736. doi: 10.1126/science.abf3736, PMID: 34140357 PMC8370718

[ref54] Zhong J. Chen D. Q. Hung P. S. Hayes D. J. Liang K. E. Davis K. D.. (2018). Multivariate pattern classification of brain white matter connectivity predicts classic trigeminal neuralgia. Pain 159, 2076–2087. doi: 10.1097/j.pain.0000000000001312, PMID: 29905649

[ref55] Ziegler G. Dahnke R. Winkler A. D. Gaser C. (2013). Partial least squares correlation of multivariate cognitive abilities and local brain structure in children and adolescents. NeuroImage 82, 284–294. doi: 10.1016/j.neuroimage.2013.05.088, PMID: 23727321

[ref56] Zikou A. K. Kosmidou M. Astrakas L. G. Tzarouchi L. C. Tsianos E. Argyropoulou M. I. (2014). Brain involvement in patients with inflammatory bowel disease: A voxel-based morphometry and diffusion tensor imaging study. Eur. Radiol. 24, 2499–2506. doi: 10.1007/s00330-014-3242-6, PMID: 25001084

