# Supplementary material for: Changes in white matter microstructure in the brain of patients with inflammatory bowel disease are associated with abdominal pain
Source: Front Neurosci. 2025 Jul 8;19:1570425. doi: 10.3389/fnins.2025.1570425 (PMC12279868; doi:10.3389/fnins.2025.1570425)
Supplement: Supplementary file 1 [file Table_1.docx]

Supplementary Material

**Table S1. **DTI metrics of significantly altered white matter tracts in HC vs. IBD and HC vs. CD comparisons****

|  | White metrics | White matter region | Left or Right | MNI coordinates | | |
| --- | --- | --- | --- | --- | --- | --- |
|  |  |  |  | X | Y | Z |
| HC vs. IBD |  |  |  |  |  |  |
| HC > IBD | MD | Anterior corona radiata | R | 73 | 168 | 105 |
|  | MD | Posterior corona radiata | R | 76 | 121 | 129 |
|  | MD | Body of corpus callosum | R | 11 | 33 | -14 |
|  | MD | Posterior limb of internal capsule | R | 63 | 105 | 85 |
|  | AD | Corticospinal tract | R | 69 | 102 | 114 |
|  | RD | Genu of corpus callosum | - | 91 | 155 | 75 |
|  | RD | Splenium of corpus callosum | - | 91 | 91 | 88 |
|  | RD | Cingulum | L | 98 | 108 | 107 |
|  | RD | Corticospinal tract | R | 82 | 103 | 43 |
|  | RD | Corticospinal tract | L | 97 | 103 | 43 |
| HC vs. CD |  |  |  |  |  |  |
| HC < CD | FA | Splenium of corpus callosum | - | 89 | 91 | 88 |
|  | FA | Anterior corona radiata | L | 105 | 163 | 67 |
| HC > CD | MD | Anterior corona radiata | R | 69 | 167 | 74 |
|  | MD | Anterior corona radiata | L | 110 | 155 | 66 |
|  | MD | Posterior corona radiata | R | 71 | 98 | 110 |
|  | MD | Posterior corona radiata | L | 110 | 98 | 112 |
|  | MD | Body of corpus callosum | - | 96 | 144 | 91 |
|  | MD | Splenium of corpus callosum | - | 90 | 89 | 88 |
|  | MD | Cingulum | R | 80 | 108 | 106 |
|  | MD | Cingulum | L | 97 | 115 | 106 |
|  | MD | Posterior limb of internal capsule | R | 63 | 106 | 88 |
|  | MD | Posterior limb of internal capsule | L | 117 | 106 | 87 |
|  | MD | Anterior limb of internal capsule | L | 100 | 129 | 71 |
|  | MD | Posterior thalamic radiation | L | 122 | 54 | 67 |
|  | MD | Fornix | - | 95 | 116 | 76 |
|  | AD | Body of corpus callosum | - | 101 | 104 | 102 |
|  | AD | Corticospinal tract | L | 97 | 106 | 45 |
|  | AD | Corticospinal tract | R | 82 | 103 | 43 |
|  | AD | Anterior corona radiata | R | 69 | 150 | 64 |
|  | AD | Anterior corona radiata | L | 105 | 157 | 60 |
|  | AD | Posterior corona radiata | R | 71 | 98 | 110 |
|  | AD | Posterior corona radiata | L | 110 | 98 | 112 |
|  | AD | Fornix | - | 93 | 118 | 73 |
|  | RD | Anterior corona radiata | R | 69 | 160 | 64 |
|  | RD | Anterior corona radiata | L | 110 | 154 | 64 |
|  | RD | Posterior corona radiata | R | 73 | 102 | 107 |
|  | RD | Posterior corona radiata | L | 106 | 117 | 107 |
|  | RD | Corticospinal tract | L | 97 | 106 | 45 |
|  | RD | Corticospinal tract | R | 82 | 103 | 43 |
|  | RD | Body of corpus callosum | - | 90 | 113 | 97 |
|  | RD | Splenium of corpus callosum | - | 91 | 145 | 90 |
|  | RD | Cingulum | R | 81 | 114 | 107 |
|  | RD | Cingulum | L | 97 | 113 | 107 |
|  | RD | Posterior limb of internal capsule | R | 62 | 108 | 91 |
|  | RD | Posterior limb of internal capsule | L | 116 | 108 | 80 |
|  | RD | Anterior limb of internal capsule | L | 103 | 133 | 76 |
|  | RD | Posterior thalamic radiation | L | 134 | 79 | 73 |
|  | RD | Fornix | - | 96 | 115 | 78 |

HC,healthy control; IBD, inflammatory bowel disease; CD, Crohn's disease; R, right; L, left.

Table S2. ****LV1-significant white matter clusters in whole-brain PLSC analysis of IBD patients****

| White matter region | Left or Right | MNI coordinates | | |
| --- | --- | --- | --- | --- |
|  |  | X | Y | Z |
| Anterior corona radiata | R | 69 | 167 | 74 |
| Posterior corona radiata | R | 71 | 98 | 110 |
| Corticospinal tract | R | 82 | 103 | 43 |
| Body of corpus callosum | - | 96 | 144 | 91 |
| Splenium of corpus callosum | - | 90 | 89 | 88 |

Table S3. Pearson correlation coefficients and statistical metrics (mean, SD, etc.) between whole - brain DTI metrics and behavioral scales.

| Mean correlation coefficient | SD of correlation coefficient | Bootstrap ratio | Two-tailed p-value |
| --- | --- | --- | --- |
| 0.105285093 | 0.091007537 | 0.3722 | 0.7094 |
| 0.256799019 | 0.089377864 | -2.3969 | 0.0165 |
| 0.257486074 | 0.094140197 | -2.7855 | 0.0053 |
| 0.113001525 | 0.089239211 | -0.1283 | 0.8980 |
| 0.155411498 | 0.08705754 | -0.2274 | 0.8201 |
| 0.259111586 | 0.086888302 | -0.6139 | 0.5392 |
| -0.405444154 | 0.077176304 | 1.1133 | 0.2657 |
| 0.806366647 | 0.033048427 | -5.6419 | 1.66e-08 |
| 0.760764148 | 0.031110454 | -2.9784 | 0.0029 |
| 0.699786398 | 0.046767165 | -4.0173 | 5.84e-05 |
| 0.833035624 | 0.026674615 | -4.6097 | 4.06e-06 |
| 0.770353763 | 0.037185545 | -4.0584 | 4.99e-05 |
| 0.627174586 | 0.094366908 | -3.5218 | 0.00043 |
| 0.69092277 | 0.043183947 | -2.4084 | 0.0160 |
| 0.621546513 | 0.056529423 | -1.7626 | 0.0779 |
| 0.756852611 | 0.037030812 | -2.6454 | 0.0082 |
| 0.52826358 | 0.116853667 | -1.7963 | 0.0726 |
| 0.635044222 | 0.057776817 | -2.5956 | 0.0094 |
| 0.61980614 | 0.079547002 | -1.8481 | 0.0647 |
| 0.56310823 | 0.071198356 | -3.7008 | 0.00021 |
| 0.283955067 | 0.092236342 | 0.0244 | 0.9805 |
| 0.278040912 | 0.084103918 | -0.2187 | 0.8270 |
